# Application of Classification Algorithms to Diffuse Reflectance Spectroscopy Measurements for Ex Vivo Characterization of Biological Tissues

**DOI:** 10.3390/e22070736

**Published:** 2020-07-03

**Authors:** Félix Fanjul-Vélez, Sandra Pampín-Suárez, José Luis Arce-Diego

**Affiliations:** Biomedical Engineering Group, TEISA Department, University of Cantabria, Av de los Castros s/n, 39005 Santander, Spain; sandra.pampin@unican.es (S.P.-S.); arcedj@unican.es (J.L.A.-D.)

**Keywords:** diffuse reflectance spectroscopy, biological tissues, tissue classification, multiple classification

## Abstract

Biological tissue identification in real clinical scenarios is a relevant and unsolved medical problem, particularly in the operating room. Although it could be thought that healthy tissue identification is an immediate task, in practice there are several clinical situations that greatly impede this process. For instance, it could be challenging in open surgery in complex areas, such as the neck, where different structures are quite close together, with bleeding and other artifacts affecting visual inspection. Solving this issue requires, on one hand, a high contrast noninvasive technique and, on the other hand, powerful classification algorithms. Regarding the technique, optical diffuse reflectance spectroscopy has demonstrated such capabilities in the discrimination of tumoral and healthy biological tissues. The complex signals obtained, in the form of spectra, need to be adequately computed in order to extract relevant information for discrimination. As usual, accurate discrimination relies on massive measurements, some of which serve as training sets for the classification algorithms. In this work, diffuse reflectance spectroscopy is proposed, implemented, and tested as a potential technique for healthy tissue discrimination. A specific setup is built and spectral measurements on several ex vivo porcine tissues are obtained. The massive data obtained are then analyzed for classification purposes. First of all, considerations about normalization, detrending and noise are taken into account. Dimensionality reduction and tendencies extraction are also considered. Featured spectral characteristics, principal component or linear discrimination analysis are applied, as long as classification approaches based on k-nearest neighbors (k-NN), quadratic discrimination analysis (QDA) or Naïve Bayes (NB). Relevant parameters about classification accuracy are obtained and compared, including ANOVA tests. The results show promising values of specificity and sensitivity of the technique for some classification algorithms, even over 95%, which could be relevant for clinical applications in the operating room.

## 1. Introduction

Biological tissue identification in the operating room is critical in many situations, particularly in tight volumetric spaces with bleeding and illumination artifacts. In these scenarios the identification cannot be reliably made by sight. Furthermore, an incorrect identification of a biological tissue can result in nerve or blood vessel resection for instance, with undesired partial paralysis or hemorrhage. It is then essential to provide healthy tissue type feedback in the operating room to avoid collateral damage.

The contributions to the solution of this problem imply a noninvasive high contrast technique, and powerful classification algorithms to provide accurate results. Regarding the noninvasive high contrast technique, optical radiation provides noninvasive, nonionizing procedures, even noncontact. Optical techniques for diagnosis of biological tissues have demonstrated great potentiality. Among others, different types of microscopy, spectroscopy [1] or fluorescence [2] have been widely used. Even tomography [3], increased contrast tomography [4] or increased contrast by polarization [5] are possible by means of advanced optical techniques. Healthy tissue classification requires a fast and high biochemical contrast technique that is relatively easy to implement, because it will probably be used in conjunction with other surgical tools. Optical diffuse reflectance spectroscopy has already shown great capabilities as a noninvasive optical technique for the study of biological tissues. Light propagation in biological tissues is strongly wavelength-dependent, and optical properties of these tissues are specific to the tissue type. The main optical properties to be considered in propagation are usually absorption ($\mu_{a}$) and reduced scattering ($\mu {´}_{s}$) coefficients, together with anisotropy of scattering and refractive index. The strong dependency of optical diagnostic techniques on optical properties is reflected in tables for different tissues that appear in the literature, either for in vivo, ex vivo or in vitro humans or animals [6,7].

A lot of studies have reported the feasibility of diffuse reflectance spectroscopy to determine the present pathological state of biological tissues, and provide information regarding tissue morphology, functionality, and/or biochemical composition. Using this technique, abnormalities in tissues can be detected, and the technique then serves as an optical biopsy tool. Optical spectroscopy has been applied for the detection of abnormal cells or cancerous tissues in different tissue types and organs, such as liver [8], bladder [9], colon [10], esophagus [11], bronchial tree [12], breast [13], brain [1], skin [14,15] epithelium [16], bone [17], muscle [18], fat [19] or nerve [20], among others. The technique has been shown to have a great potential as an inspection and diagnosis procedure. Spectroscopy does not require ionizing energy that harms the tissue, as previously said, and can be implemented in optical fiber, characteristics that greatly facilitate the development of a clinical instrument.

The clinical studies just described use optical systems to illuminate the particular organ or tissue under study, and collect the spectral characteristics of backscattering light for a particular wavelength range. According to the usual application of distinguishing pathological, mainly tumoral, and healthy tissues, the diagnostic analysis is based on two types of classes, either healthy or pathological tissue. The distinction implies in the end a typical class discrimination problem [21]. Apart from other considerations, this kind of analysis usually requires a large number of measurements, also from different specimens, in order to report statistically significant conclusions.

The problem that is addressed in this work is slightly different, as we are interested in healthy tissue discrimination, so as to be sure of the tissue type that is in front of the surgical tool in the operating room. Previous studies have shown the possibility of this analysis for some tissue types [21], and even specifically for adipose tissue, that intraclass variability can be an issue in the classification problem depending on the deviation of the measured specimens and experimental conditions [22], or the influence of experimental conditions [16]. However, a deep analysis of several classification algorithms that try to address the problem of identifying more tissue types, mainly bone, muscle, nerve, fat or skin is missing. This is an unsolved clinical problem, and needs a reasonably simple and affordable technique to be implemented in a clinical device. In order to test this possibility, first of all ex vivo biological tissues of different kinds are obtained. Afterwards, a specific optical setup is built in order to measure the spectra. The measured spectra present high dimensionality, which makes the classification problem difficult to cope with. Several approaches were implemented in order to extract relevant characteristics that could be afterwards introduced into final classification algorithms to test the discrimination power. From all of them, one based on characteristics extraction, specifically particular gradients according to spectral characteristics, and another more general one based on principal component analysis are shown. The accuracy results demonstrate the potential of diffuse reflectance spectroscopy for healthy tissue discrimination, an issue of critical relevance in several clinical interventions. For example, neck surgery could greatly benefit from this approach, as failure to distinguish a blood vessel or a nerve that are finally resected could have great negative consequences on the patient.

In this work, ex vivo porcine biological tissues of several types are extracted, adequately processed, and measured by a diffuse reflectance spectroscopy setup. The optical properties of porcine biological tissues have been shown to be near to human [23], so the approach could be extended to clinical applications. The samples come from different specimens, and are obtained on different days. Several spectra are captured from each sample, so as to reduce noise and nontissue type dependent variability. Spectral measurements represent high dimension data, and as a consequence the classification problem is computationally complex. Several approaches, based on characteristics extraction or components extraction, mainly principal components analysis (PCA), are employed. Afterwards classification approaches based on k-nearest neighbors (k-NN), linear discriminant analysis (LDA), quadratic discriminant analysis (QDA) or Naïve Bayes (NB) are applied. The results from these approaches are compared by statistical analysis such as ANOVA, and conclusions are extracted regarding the best approach for a potential clinical application.

Section 2 of this article describes the materials and methods employed. It first shows the details of the biological porcine samples and processing approaches. Afterwards the experimental setup for diffuse reflectance spectroscopy is described. The criteria for obtaining the spectra are also exposed. The algorithms for data dimensionality reduction, noise reduction or misalignment corrections are also described. Section 3 presents the results of analyzing the measured spectra by means of several approaches, mainly characteristics extraction and principal component analysis. The particular characteristics are described, applied, and evaluated, including the classification algorithms for class assignment. The same approach is implemented with the principal components, that are further classified and evaluated. The statistical approaches for the evaluation are described and applied. Section 4 contains the conclusions of the work.

## 2. Materials and Methods

### 2.1. Ex Vivo Samples and Experimental Setup

**Figure 1:**
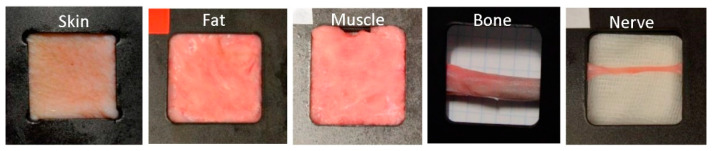
Examples of some of the porcine samples extracted in the specifically designed holders, that shows the open imaging window. From left to right, skin, fat, muscle, bone, and nerve.

Ex vivo porcine samples were obtained from healthy Large White pigs, with the prior approval of the Bioethics Committee of the Valdecilla Virtual Hospital (Santander, Spain). The specimens were between 2 and 4 months old, with a weight of between 20 and 25 kg each. Skin samples were extracted from the upper inner leg, fat tissue from the neck, muscle tissue from the leg, bone from the rib and nerve from the sciatic nerve. At least two samples of each tissue were extracted from each of the ten specimens. According to the approved experimental procedure, sample extraction was performed by a trained veterinary surgeon. Special attention was paid to avoid mechanical damage in the extraction process. A specifically designed sample holder was employed to fix the samples. The sample holder leaves exposed an area of approximately 3 × 3 cm^2^, enough for diffuse reflectance measurements. Examples of the samples in their holders are shown in Figure 1.

Immediately after extraction every sample was gently rinsed with sodium chloride at 9 mg/mL to clean and maintain sample humidity. Afterwards they were wrapped with gauze that was previously rinsed with sodium chloride. The samples were stored at 10˚C, with a maximum of 12 hours before measurements beginning. 

**Figure 2:**
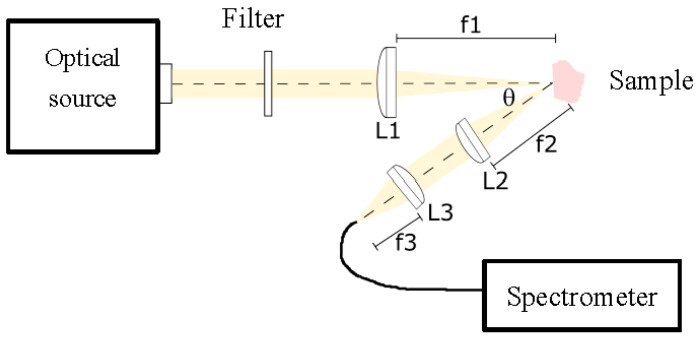
Experimental setup for diffuse reflectance spectroscopy. A white optical source is filtered for UV blocking. A lens system L1 focuses light onto the sample. This light is collected by a lens system L2−L3 at an angle of approximately 30°, and finally focused on an optical fiber to the spectrometer.

The experimental setup built and employed in this work for diffuse reflectance spectroscopy is shown in Figure 2. This setup comprised a white light source, with a 250 W QTH lamp (Newport Corporation), that is capable of providing optical radiation with reasonable irradiance in the range between 200 and 2400 nm. The spectrum is relatively flat in the range from 550 to 700 nm, with slight smooth decays below and above this range. The optical beam was then first filtered for UV radiation elimination, as it could be potentially ionizing for this medical application. Afterwards the beam was collimated and focused by a fused silica lens system described in the scheme as L1. The size of the spot was about 3 mm in diameter on the tissue sample. This beam diameter was in accordance with tissue sample sizes, particularly with those of bone and nerve tissues, in such a way that the optical beam was always impinging completely on part of the tissue sample, and it never went beyond it. We did not employ a smaller beam size as we tried to imitate clinical praxis, where an optical probe would be used, at arbitrary distances from the tissue and, as a consequence, with probable extended spot diameters. Although it would be better, from the classification point of view, to have a smaller spot size, that scenario would not be in accordance with the expected clinical use. In this more realistic scenario, we could in principle assure that, if the classification worked in this situation, it would work for any other smaller spot sizes, until the signal to noise ratio degrades the quality of the spectra. In the present work we were far away from this limitation. The tissue sample was vertically positioned on a micrometric stage, so it could be displaced in any dimension with a step of 10 μm. Light was then diffusely reflected from each sample. A collecting arm was designed to capture diffuse reflected light at an angle θ of 30°, in order to avoid specular reflection, in line with the literature. We made some experimental measurements with other angles, either smaller or bigger, and found a compromise between specular reflectance and enough signal power around this angle. The optical collection system was represented in the graph with lenses L2 and L3, and consisted mainly on a collimating and focusing system. The acceptance cone was aligned with the illumination spot, in order to assure that captured radiation belonged to the illuminated area. The final focusing step concentrated collected light onto a multimode 1 mm core diameter optical fiber, with a broadband optical window and solarized. This optical fiber was directly connected to a spectrometer (StellarNet Inc., Tampa, FL, USA). The spectrometer was based on a CCD and provided a spectral range from 220 to 1100 nm with a resolution of 0.5 nm. The control software allowed the adjustment of the integration time, spectral and temporal averaging, and background spectrum. The complexity of the optical system was designed to be as simple as possible, first of all for reducing the overall cost of a clinical device, and secondly due to the fact that probably the distal part of the device should be discarded after use to prevent biological contamination from patient to patient.

The reflectance spectra were computed by first capturing the reflected spectrum of a reference standard (StellarNet Inc., Tampa, FL, USA) that was able to diffusely reflect light in a range from 250 to 1500 nm. All measurements were made by subtracting a background spectrum measured by blocking the optical source. Afterwards the sample was located in place of the reference standard, and the spectrum was measured. The relationship between the spectrum of the sample and that of the reference standard was the reflectance measurement.

Temporal averaging of 10 spectra was employed to reduce random noise, as well as spectral averaging to avoid outliers. A total of 4 measurements were obtained at each point. A total of 16 points were measured on each sample, trying to maintain a nonoverlapping approach with constant distance. Displacements were made by the micrometric stage. A two-dimensional displacement was implemented, except for bone and nerve, that due to their geometry required just a horizontal displacement. A total of 6400 spectra were obtained.

### 2.2. Spectra Preprocessing

**Figure 3:**
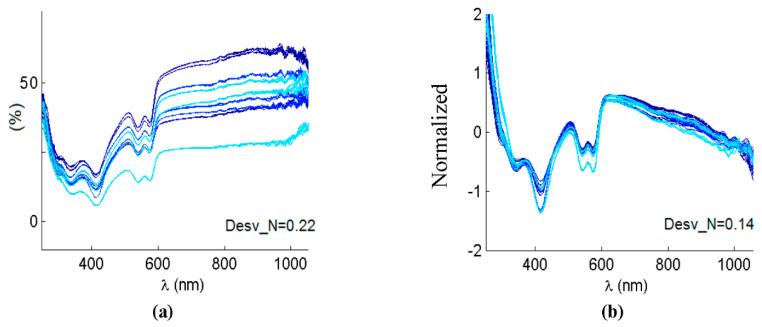
(**a**) Several original reflectance spectra. (**b**) The same spectra after applying normalization and linear detrend. Desv_N is the average difference from the linear regression fit before and after detrending.

Once the spectra were measured as previously stated, several steps were followed to preprocess data before applying further classification analysis. As data came from different specimens and from different days, so it would correspond to a real clinical situation, some previous steps helped to remove systematic scattering noise. This effect could be based on instrument artifacts, or even on characteristics of the sample itself, such as surface rugosity or exact orientation, among others. A first step dealt with spectra normalization, that was implemented by means of subtracting the mean of the spectra, and by adjusting the standard deviation of the data to unity. Apart from that, and as the referred-to undesired effects usually manifested themselves as linear trends, a detrend filter was applied [24]. An example of original vs. normalized and after-detrend spectra is shown in Figure 3. A trend to increase can be clearly appreciated in Figure 3a, along with mean and standard deviation differences, which could greatly affect the results. Figure 3b shows the same spectra after normalization and linear detrend algorithms. All the spectra were now aligned, had null mean and unity standard deviation.

Noise filtering was also implemented, in this case by means of a Savitzky–Golay filter [25]. This filter is able to retain the relevant characteristics of the spectra, mainly peak positions, and width, that are the potential parameters that will allow tissue classification.

Finally, as the system was based on a spectrometer at the end, it was very important to maintain an adequate calibration of the device. This was particularly critical in the spectral line calibration, as a misalignment could artificially deviate peak positions, and these positions were understood as fundamental from the knowledge of optical interaction from biological tissues. Although the spectrometer was calibrated as required, thinking also on the final application where this could not be the case, alignment algorithms were included, mainly dynamic time warping and icoshift [26]. 

## 3. Results

**Figure 4:**
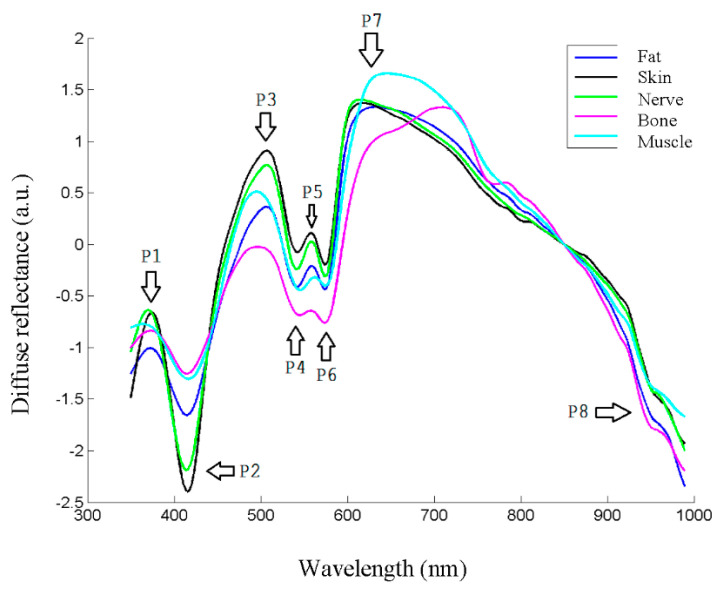
Reflectance spectra of different tissue types, where main peaks and valleys are marked as P1–P8.

The spectra were collected as previously stated from the extracted samples, and the preprocessing steps were carried out. The appearance of typical spectra, coming from each of the samples, is shown in Figure 4. As in the usual spectroscopic measurements, the locations of peaks and valleys are particularly relevant, because they are related with biochemical information from the sample. In the case of biological tissues, hemoglobin, water or even proteins or other pigments can dominate the spectral response [27]. Hemoglobin peaks and valleys are usually around 425 and 555 nm for the deoxygenated state, and around 410, 540 and 575 for the oxygenated one [7], proteins present another peak at 280 nm [28], water shows peaks in the IR at 970 or 1197 nm [29], and lipids at 930 or 1210 nm [19]. Usually spectra coming from biological tissues present a decrease that starts around 650 nm [30].

As can be seen in Figure 4, there seemed to be differences between the biological tissue types, but they were not so evident by sight as by looking at the spectra. In fact, the location of most peaks and valleys was common to all of them, as previously said regarding the main components that influence the spectra, which most of the tissues contained. For instance, the presence of peaks or valleys at 415 or 575 nm could be attributed to deoxygenated hemoglobin, as they were ex vivo samples, or the 970 nm valley to water, present also in all the tissues. 

Therefore, it was clear that classification analysis was needed in order to try to provide further classification information for the intended application. One of the first issues to be solved had to do with the high dimensionality of the data, which could make the computation of classification algorithms difficult. As not all the spectral information seemed to provide information for tissue classification, as just discussed, dimensionality reduction would be applied. Two main initial approaches would be carried out. The first one would be based on spectra characteristics extraction, while the second one would start from PCA. After the dimensionality of the problem was reduced, classification algorithms would be applied, and statistical conclusions would be extracted.

### 3.1. Classification Algorithms Based on Characteristics Extraction

Several tests were made with different characteristics from the spectra, and the conclusions showed that gradients based on spectral characteristic points were the most significant ones. The position of peaks and valleys on the spectra is then critical for adequately defining the gradients. Table 1 contains the specific nine points considered in the analysis. These points were found in each particular spectrum and taken as the references for the gradients. Null first derivative implied that the first derivative of the curve was calculated, and afterwards a point of zero value was looked for in the vicinity of the showed wavelengths.

**Figure 5:**
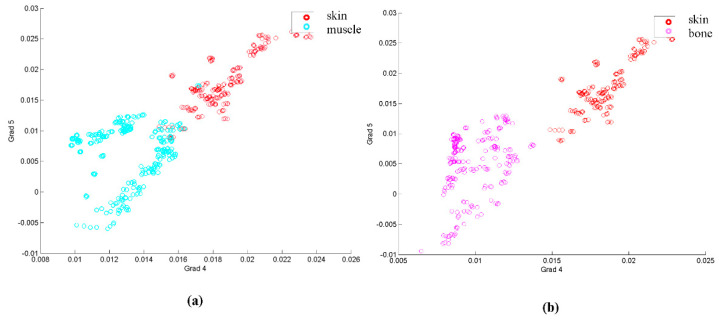
(**a**) Dispersion diagram based on gradients between points 4 and 5 and between points 5 and 6 for skin and muscle. (**b**) Same dispersion diagram for skin and bone.

In order to take into account the evolution of these points, the analysis was made with the gradients of the curve formed by the previously mentioned points at their respective wavelengths. A first visual analysis employed a dispersion diagram with two of the gradients, the one relating points 4 and 5, and the other one joining points 5 and 6, for three tissue types, skin, muscle and bone. This diagram appears in Figure 5. 

Looking at Figure 5, it seems that, at least for the example shown, several samples of some of the classes could be classified by these two gradients, as they are separated in the diagram. However, the classification approach needed a whole classification for all the tissues, and also statistically significant parameters for comparison.

**Figure 6:**
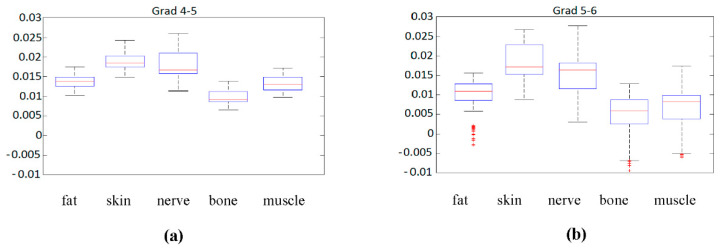
(**a**) Boxplot of gradient 4 to 5 for the different tissue types. (**b**) Boxplot of gradient 5 to 6 for the different tissue types.

The first statistical analysis that could be made is by means of a boxplot diagram. This boxplot represented the median of each parameter for each sample according to its class (tissue type), as long as the first and second quartile, and the maximum and minimum values. Figure 6 contains this information for the previously analyzed gradients 4–5 and 5–6.

Although gradient 5 to 6, Figure 6b, seems to be more significantly spread in the sense of median, it also presents larger variations in the same class, and several outliers that could complicate the classification. Regarding gradient 4 to 5, Figure 6a, the spread of data values is more reduced, but the median values seem to be closer to each other, something that could complicate classification.

**Figure 7:**
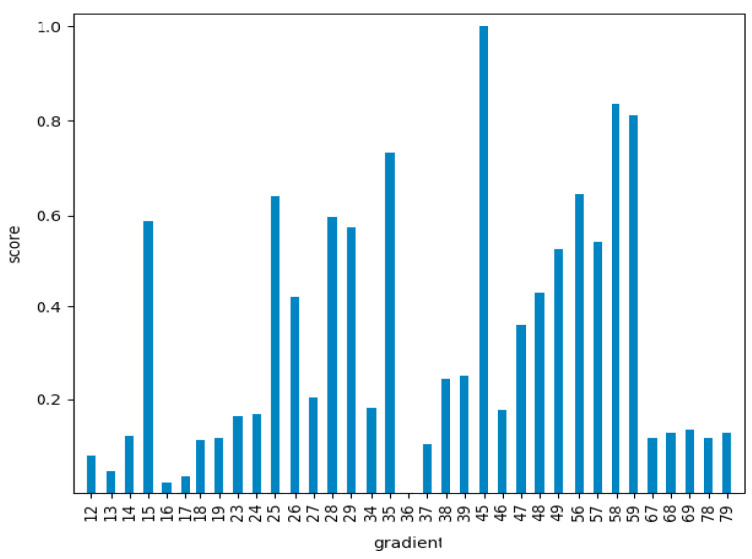
Snedecor F values for all the characteristic gradients considered, normalized by the maximum value for gradient 4–5 (663.6).

To statistically quantify these impressions, an ANOVA analysis was performed. In this analysis the mean values of each class were compared, taking their equality as the null hypothesis of the test. The Snedecor F parameter, that should be significantly high if the null hypothesis is false (that is, if the mean values of each class are statistically different) was calculated for all the gradients. The results of the F Snedecor, normalized by the maximum value, appear in Figure 7.

As can be seen in Figure 7, there were significant differences between the different gradients. This meant that the ones with lower values would not be quite appropriate for the classification problem, as the mean values of the different classes would be statistically similar. Nevertheless, the results of this test gave us information about the general classification potential, but not about the particular classification accuracy for each particular class. The analysis of this information required classification algorithms as a final step.

Five different classification algorithms were applied to the gradients, either including all of them (81), or just the first 14 with the larger F according to Figure 7. One fifth of the data was used for training the algorithm, and the rest were employed as test data for obtaining the results. Several final classification algorithms were employed. Linear discriminant analysis (LDA) divides the input domain into several regions, defined by linear hyperplanes. This algorithm shows the best performance when the input data are linearly separable, and can be formulated as:

(1) $$y_{k}\left(\vec{x}\right)={\vec{w}}_{k}^{T}\;\vec{x}+w_{k0}$$

In this equation ${\vec{w}}_{k}^{T}$ is the weight vector and $w_{k0}$ is the bias for each class. A point is assigned to a class if $y_{k}\left(\vec{x}\right)$ is bigger for the class k, when compared with all the other classes. If the discrimination boundaries are allowed to be of higher order, or curves on the graph, the algorithm is called quadratic discriminant analysis (QDA). It is also possible to try to finally classify data by a Naīve Bayes (NB) algorithm, in which statistical independence is assumed between the characteristics employed for the classification. Each input is then classified in order to maximize the following expression or the probability of multidimensional data $\vec{x}$ belonging to class $C_{k}$:

(2) $$p\left(C_{k}|x_{1},\ldots ,x_{N}\right)=p\left(C_{k}\right)\overset{n}{\underset{i=1}{\prod }}p\left(x_{i}|C_{k}\right)$$

k-nearest neighbors (k-NN) algorithm classifies an input based on the previous classification of k inputs that are near it in the space domain, usually by a constant distance criterion. The input is classified according to the maximization of the following probability, that is the ratio of the number of neighbors that belong to k class $K_{k}$ to the total number of neighbors K:

(3) $$p\left(C_{k},\vec{x}\right)=\frac{p\left(\vec{x},C_{k}\right)p\left(C_{k}\right)}{p\left(\vec{x}\right)}=\frac{K_{k}}{K}$$

Classification and regression trees (CART) divide the classification space according to boundaries that are parallel to the main axes of the space. These dimensions are segmented and form a tree structure, by means of which each input goes along the tree until it reaches a node. The structure of the tree is built according to a maximization of the *p*-value of the predictor.

The first algorithm tested was LDA, whose confusion matrix is the following:

(4) $$Mc_{LDA}=\left(\begin{array}{ccccc} 249 & 4 & 6 & 21 & 0\\ 27 & 195 & 10 & 0 & 0\\ 26 & 6 & 245 & 3 & 0\\ 49 & 2 & 0 & 224 & 29\\ 12 & 0 & 0 & 43 & 161 \end{array}\right)$$

The obtained values for sensibility and specificity for each tissue type appear in Table 2.

A similar analysis was performed with QDA, kNN, CART and NB. The results for the classification accuracy when using the best 14 gradients, defined as the ratio between correctly classified samples and the total number of samples, appear in Table 3.

From Table 3 it could be seen that the best classifier was kNN, followed by CART, with almost a 95% accuracy for the spectra employed. From the results it seemed that the gradients of the different classes (or type tissues) did not accomplish with a linear LDA or quadratic QDA model, or at least not so well as they did with adjacent measurements of neighbors (kNN) in the gradients space. A CART model was quite near in accuracy to the best kNN approach, which made sense as this approach was specifically optimized for p-value. The low accuracy of NB could be expected, as the assumption of independence of the gradients was not necessarily true. 

### 3.2. Classification Based on Principal Component Analysis 

As previously stated, another alternative to dimensionality reduction is PCA [31,32]. The PCA algorithm transforms a group of possibly correlated variables into a number of equal or smaller, uncorrelated, or orthogonal variables. These transformed variables are called principal components. Let X be an N×D matrix whose rows are the N observations. Each observation or spectrum $\left\{{\vec{x}}_{n}\right\}$ belongs to a D-dimensional space. The algorithm projects the data on a space of lower dimension while the variance of the projected data is maximized. Let ${\vec{u}}_{1}\in {\mathbb{R}}^{D}$ be a unit vector which defines the direction of the new space. The projected data variance can be expressed as:

(5) $$\frac{1}{N}\sum_{n=1}^{N}{\left\{{\vec{u}}_{1}{}^{T}{\vec{x}}_{n}-{\vec{u}}_{1}{}^{T}\bar{x}\right\}}^{2}={\vec{u}}_{1}{}^{T}S\;{\vec{u}}_{1},$$

where $\bar{x}$ is the mean of the dataset, S the data covariance matrix and superscript T denotes transpose. The maximization of expression (5) is done with respect to ${\vec{u}}_{1}$, defining the following constraint ${\vec{u}}_{1}{}^{T}{\vec{u}}_{1}=1$. It can be demonstrated that optimal projection is obtained when the new space is defined for the first eigenvectors $\left({\vec{u}}_{1},{\vec{u}}_{2},\ldots ,{\vec{u}}_{M}\right)$ of the covariance matrix S.

**Figure 8:**
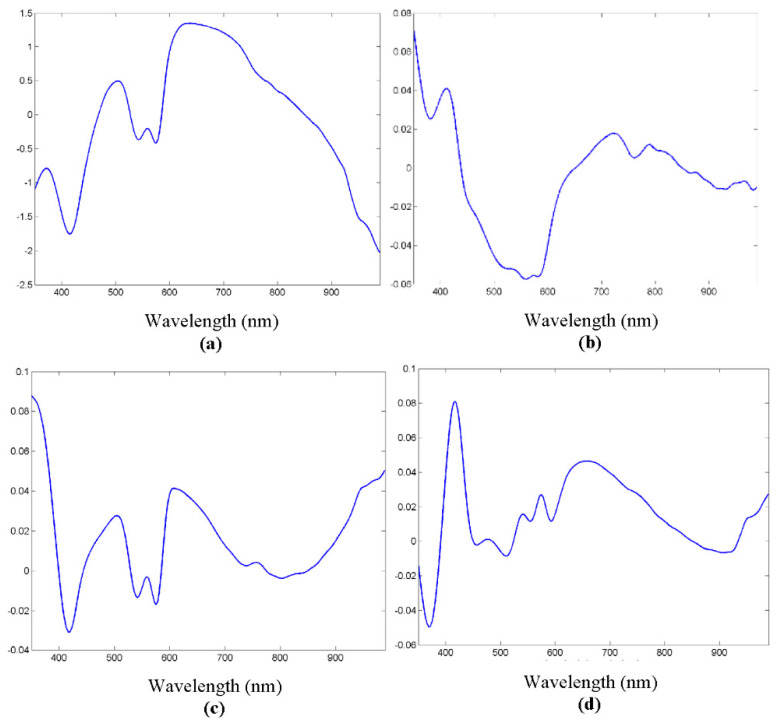
(**a**) Original spectrum example. (**b**) First principal component analysis (PCA) component. (**c**) Second PCA component. (**d**) Third PCA component.

Figure 8 shows the results of the first three components of the PCA. It could be seen that several peaks and valleys were reinforced, as they were supposed to contribute to signal variability between tissue types.

The number of principal components needed for adequate spectrum reconstruction, that is, for significant signal information, is so that the first component presents on average around 82% similarity, while with just 40 components the similarity increases to over 99%. This makes the dimensionality reduction obvious, as just the desired number of components need to be used, instead of the whole spectrum.

**Figure 9:**
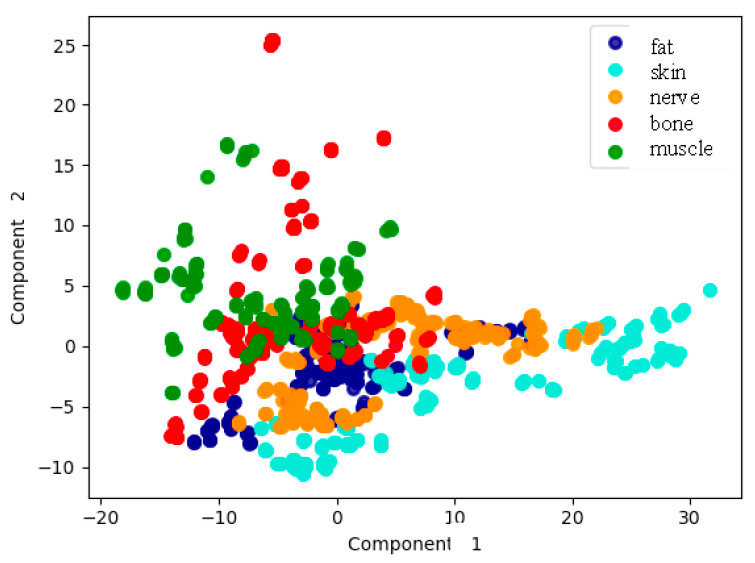
Representation of the measurements according to tissue type, based on the first two PCA components.

Figure 9 shows the results of just the first two PCA components as a function of tissue type. Although it was true that several tissue types were not clearly distinguished, the potentiality of the analysis with only two components seemed promising.

As in the previous case regarding characteristic parameters in Section 3.1, the same classification algorithms, LDA, QDA, kNN, CART and NB, are applied to only the first 40 PCA components. In the case of the application of LDA, the confusion matrix is as follows:

(6) $$Mc_{LDA}=\left(\begin{array}{ccccc} 279 & 0 & 0 & 1 & 0\\ 1 & 231 & 0 & 0 & 0\\ 1 & 0 & 279 & 0 & 0\\ 3 & 0 & 0 & 301 & 0\\ 0 & 0 & 0 & 2 & 214 \end{array}\right)$$

In this case, again for LDA the values of specificity and sensibility appear in Table 4.

These results clearly show a great classification power of the discriminant. As in the previous case of characteristics, other classification algorithms were applied, and the accuracy results are shown in Table 5.

Large differences in the accuracy can be appreciated in Table 5, although several classification algorithms are over 95%. The results imply a great accuracy of the results from these data.

## 4. Discussion

In this work two main classification approaches have been implemented and analyzed, one based on characteristics extraction, and the other one on principal component analysis. Both of them were intended for dimensionality reduction, as the large amount of data in each spectrum makes it difficult to provide an easily computable approach. Several classification approaches have been tried for each alternative.

The results with the first option, the one regarding spectra characteristics, has been focused on relevant peaks and valleys in the spectra, as suggested by the knowledge of the main biochemical components of the biological tissues, that usually condition spectroscopic measurements. This prior information is supposed to increase the efficiency of the approach. As said, not only these points but the gradients they form are considered. According to the first proposed data, a total of 81 gradients should be included in the analysis. As these points must be automatically looked for, by calculating the first derivative of the spectrum, and the analysis must be made with 81 values for each spectrum, it can be computationally intensive. The analysis concluded that just 14 gradients were the most significant ones for the analysis, greatly reducing the dimensionality of the problem. The subsequent application of classification algorithms, whose main results are in Table 3, showed a maximum accuracy of almost 95% for the k-NN approach. The next one was CART, with around 93%, and afterwards came LDA, that dropped to almost 82%. A classification accuracy around 95% seemed to be quite promising then for the final application.

Regarding the second option, based on principal component analysis, the results showed that it was possible to employ just 40 components in order to have very significant results. PCA is a quite common algorithm that can be relatively easily implemented. Conversely to the previous approach, the selection of the data is made by purely mathematical reasons, and not by prior knowledge of biochemical components in the tissues. This fact could make the procedure more reliable perhaps in a situation with unknown different samples or other elements that alter the spectra of the samples. The application of the same classifiers as in the previous case, as it appears in Table 5, gave accuracies of even more than 99% for QDA and LDA approaches, while kNN and CART were over 94%, and only NB dropped to 80%. The results were then better than those of the previous approach, as an almost perfect classification seemed to be possible. The interest of the results relied on final accuracy classification measures of massive measurements that demonstrated the potential feasibility of the technique for the clinical application of healthy tissue discrimination.

Of course, the results are very promising for solving the issue that was exposed at the beginning, based on the fact that discriminating tissue types is a clinical need. However, and despite the results, some considerations for clinical translation should be made. On one hand, the samples were specifically extracted and treated, so as to assure a high purity regarding tissue type, which would allow good classification algorithm training. In real clinical setups, there would be occasions in which more than one tissue type are in front of the operating area, and the response of the algorithms would probably change. Nevertheless, an appropriate classification would first need the analysis made in the present work. The spectra measured in this work constitute a core part of any classification algorithm of multilayered biological tissues, as they are obtained from known samples of one specific tissue type. Monte Carlo simulations regarding penetration depth and volumetric performance, as a function of tissue type and wavelength, show penetration depths of between 1 and 2 mm. Several relevant tissue types of interest in clinical practice are of that order of magnitude or even thicker. A precise model of the reflectance spectrum, even including a Lambertian model of the emission, could maybe add more relevant information for the classification. However, such a model would rely on specific assumptions of distance to the sample, angular incidence, and biological tissue structure, which we could not assure in the clinical praxis by an optical probe with uncontrolled distances to the sample. The denomination diffuse reflectance spectroscopy is quite extended in the field for spectral reflectance measurements, although sometimes the measured spectra might not follow strictly a theoretical diffuse model. A complete experimental analysis of multilayered tissue classification would be the next step in this work. On the other hand, several other components, such as additional blood, rinse water or other surgical elements could be in the operating area at the time of the measurement, and those elements would definitely alter the measured spectra. All these considerations should be taken into account in the next steps, as they would for sure decrease the classification accuracy.

The main aim of the manuscript regarding the clinical application is to provide first a simple and affordable tool for timely tissue identification, by means of a fiber probe. This would allow the surgical cutting procedure even to automatically stop when an undesired biological tissue is in front of the instrument, such as a nerve or a blood vessel. The technique could be extended to 2D measurements if we would like to obtain a tissue type map of the area of interest. That scenario would be possible by a fiber scanner, on one side, or even by a multispectral camera. Both approaches present challenges, as a fiber scanner could be expensive, difficult to stabilize and not fast enough, and a multispectral camera is expensive and provides limited spectral resolution.

## 5. Conclusions

Diffuse reflectance spectra have been measured with a specific setup built at the lab. Samples were extracted from ex vivo Large White pigs, and systematically measured. Each spectrum was normalized, detrended and filtered for noise reduction before the classification. Two main approaches have been used, one based on spectral characteristics, and the other one on principal component analysis for dimensionality reduction. Several classification analyses have been applied and evaluated. Accuracies of even 99% have been shown to be possible in the experimental conditions of this article, so diffuse reflectance spectroscopy has been proved to be potentially useful in clinical applications of healthy tissue discrimination. Of course, clinical translation of the results needs the consideration of other practical issues, such as impure samples or the presence of additional components in the surgical area, that must be faced in the next steps.

## Figures and Tables

**Table 1:** The nine characteristic points of the spectra.

Point	Type	Main Biological Compound
**1**	Minimum at 410 nm	Hemoglobin
**2**	Maximum at 450–540 nm	Hemoglobin
**3**	Minimum at 540 nm	Hemoglobin
**4**	Maximum at 545–570 nm	Hemoglobin
**5**	Minimum at 575 nm	Hemoglobin
**6**	Null first derivative at 940–970 nm	Water
**7**	Null first derivative at 436 nm	Hemoglobin
**8**	Null first derivative at 934 nm	Water
**9**	Null first derivative at 958 nm	Water

**Table 2:** Linear discriminant analysis (LDA) results for each tissue type from characteristics extraction.

Tissue Type	Specificity	Sensitivity
**Fat**	0.69	0.89
**Skin**	0.94	0.84
**Nerve**	0.94	0.88
**Bone**	0.77	0.74
**Muscle**	0.85	0.75
**Average**	0.83	0.82

**Table 3:** Accuracy results for each classifier from characteristics extraction.

Classifier	Accuracy
**LDA**	0.818 ± 0.036
**QDA**	0.807 ± 0.040
**kNN**	0.949 ± 0.024
**CART**	0.932 ± 0.019
**NB**	0.668 ± 0.027

**Table 4:** LDA results for each tissue type from PCA analysis.

Tissue Type	Specificity	Sensitivity
**Fat**	0.98	1.00
**Skin**	1.00	1.00
**Nerve**	1.00	1.00
**Bone**	0.99	0.99
**Muscle**	1.00	0.99
**Average**	0.99	0.99

**Table 5:** Accuracy results for each classifier from PCA analysis.

Classifier	Accuracy
**LDA**	0.993 ± 0.005
**QDA**	0.998 ± 0.003
**kNN**	0.986 ± 0.014
**CART**	0.949 ± 0.016
**NB**	0.803 ± 0.028
